# Phenolic profiles and antioxidant activities in selected drought-tolerant leafy vegetable amaranth

**DOI:** 10.1038/s41598-020-71727-y

**Published:** 2020-10-26

**Authors:** Umakanta Sarker, Shinya Oba

**Affiliations:** 1 Department of Genetics and Plant Breeding, Faculty of Agriculture, Bangabandhu Sheikh Mujibur Rahman Agricultural University, Gazipur, 1706 Bangladesh; 2 Laboratory of Field Science, Faculty of Applied Biological Sciences, Gifu University, Yanagido 1-1, Gifu, Japan

**Keywords:** Biochemistry, Natural variation in plants

## Abstract

Four selected advance lines of drought-tolerant leafy vegetable amaranth were characterized for phenolic profiles, vitamins, and antioxidant activities. The selected advance lines exhibited differences in terms of genotypes with remarkable phenols, vitamins, flavonoids content, and potential radical quenching capacity. We identified twenty-five phenolic and flavonoid compounds including protocatechuic acid, salicylic acid, gentisic acid, gallic acid, β-resorcylic acid, vanillic acid, *p*-hydroxybenzoic acid, chlorogenic acid, ellagic acid, syringic acid, ferulic acid, kaempferol, *m*-coumaric acid, *trans*-cinnamic acid, quercetin, *p*-coumaric acid, apigenin, caffeic acid, rutin, sinapic acid, isoquercetin, naringenin, myricetin, catechin, and hyperoside. The selected advance lines VA14 and VA16 had abundant phenols, vitamins, flavonoids, and antioxidants potentiality. The selected drought-tolerant leafy vegetable amaranth showed high antioxidant potentiality as phenols, vitamins, flavonoids of these lines had a significant positive correlation with antioxidant capacities equivalent to Trolox using 2,2-diphenyl-1-picrylhydrazyl and ABTS^+^*.* Therefore, drought-tolerant leafy vegetable amaranth VA14 and VA16 can be grown in semi-arid and drought-prone areas in the world to attaining vitamins and antioxidant sufficiency. The phenolic and flavonoids compounds identified in drought-tolerant leafy vegetable amaranth demand a comprehensive pharmacological study. The baseline data on phenolic and flavonoids compounds obtained in the present study will contribute to the scientist forum for the scientific evaluation of these compounds in vegetable amaranth.

## Introduction

The edible baby leaves and fleshy juvenile stems of vegetable amaranth contain high dietary fiber, protein including lysine and methionine^1–3^, minerals^4,5^, vitamins^6–8^, carotenoids, abundant pigments^9–11^, phenolic and flavonoid compounds^12–15^. It is widely distributed in Africa, Asia, America, Australia, and Europe. As these compounds have high reactive oxygen species (ROS) quenching capacity, these compounds remarkably contributed to the industry of food and protect many diseases including arthritis, cardiovascular diseases, cancer, emphysema, cataracts, retinopathy, atherosclerosis, and neurodegenerative diseases^16–20^. Hence, vegetable amaranth has a crucial role in health-promoting effects and as natural preservatives of food products^21^. Vegetable amaranth is tolerant of drought stress^22–25^ and salinity^26–28^.

Vegetable amaranth has great diversity in the Asian continent including Bangladesh, India, and South East Asia^29^ with multiple uses. It is a low-cost and important leafy vegetable in the Asian continent including Bangladesh, India, and South East Asia with the lucrative color of the leaf, taste, and abundant nutritional value. Vegetable amaranth is grown year-round as well as in the gaps of foliage crops between winter and hot summer^1,2^. Vegetable amaranth leaves especially *A. tricolor* inhibited the proliferation of breast (MCF-7), colon (Caco-2) cancer cell lines, and liver (HepG2) and exhibited anticancer potential^30^.

In this decades, food researchers and pharmacologists are interested in plant phenols and flavonoid compounds, availability in diets, their antioxidant potentiality, and roles of preventing deadly diseases including neurodegenerative, cardiovascular diseases, and cancer^31^. Antioxidants are available in vegetables i.e., phenols, flavonoids, and vitamins protect many chronic diseases^32^. Phenolic components are available in plant classified as phenols (hydroxycinnamic acids and hydroxybenzoic acids), flavonoids, tannins, and lignins that are responsible for lucrative color, antioxidant potentiality, flavor, bitterness, odorness, and acerbic taste^33^. In the human body, antioxidants compounds prohibit the oxidizing chain reactions of free radicals in molecules and reduce oxidative damage^34^. The shikimic acid pathway of plant cells plays a significant role to convert tyrosine and phenylalanine into phenols and flavonoid compounds^35^. In the human body, phenols and flavonoids have remarkable biological functions. Quercetin protects the oxidation of low-density lipoprotein through quenching free radicals in the body^36^. Ellagic acid has considered as important health-promoting compounds due to its anticarcinogenic and antimutagenic responses^37^.

However, the phenols, vitamins, and flavonoids compounds in drought-tolerant leafy vegetable amaranth have not been studied. Currently, we are examining the possibility of utilizing phenols, vitamins, and flavonoids compounds of leafy vegetable amaranth, as it has abundant good natural antioxidants of interest in the pharmacology and food industry^18,20^. Previously, forty-three vegetable amaranth genotypes were screened based on yields, drought-tolerant, and antioxidant activity to select the best four drought-tolerant, high yielding, and potential antioxidant enrich genotypes VA14, VA11, VA6, and VA16. It is the first attempt to study the phenols, vitamins, and flavonoids compounds in drought-tolerant leafy vegetable amaranth. Therefore, we characterized in detail the phenols, flavonoid compounds, and evaluate vitamin contents as well as antioxidant potentiality in drought-tolerant leafy vegetables using HPLC and LC–MS. The findings explore the understanding of phenols, vitamins, flavonoids compounds, and antioxidant potentials of drought-tolerant leafy vegetable amaranth for the pharmacists, food industry, consumers, and nutritionists.

## Results and discussion

The analysis of variance revealed a wide range of variability of the studied traits regarding selected drought-tolerant leafy vegetable amaranth. A wide range of variability was also reported in red and green color amaranth^11^, rice^38–50^, and maize^51–53^.

### Flavonoids and phenolic acids

**Figure 1:**
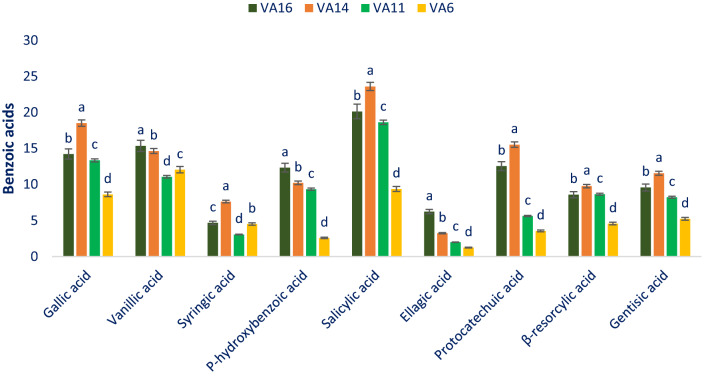
Benzoic acids profiles (µg g^−1^ FW) in selected four drought-tolerant leafy vegetables amaranth, different letters in the bar are differed significantly by Duncan multiple range test ((P < 0.01), (n = 3)).

**Figure 2:**
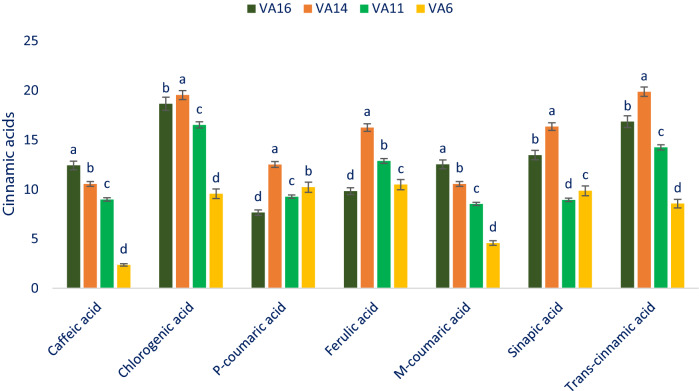
Cinnamic acids profiles (µg g^−1^ FW) in selected four drought-tolerant leafy vegetables amaranth, different letters in the bar are differed significantly by Duncan multiple range test ((P < 0.01), (n = 3)).

**Figure 3:**
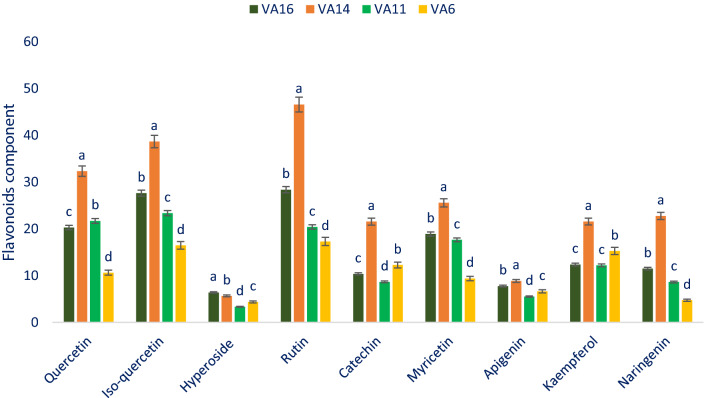
Flavonoids profiles (µg g^−1^ FW) in selected four drought-tolerant leafy vegetables amaranth, different letters in the bar are differed significantly by Duncan multiple range test ((P < 0.01), (n = 3)).

**Figure 4:**
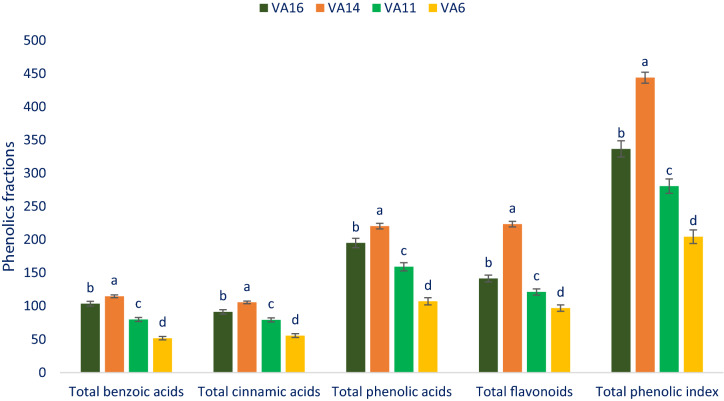
Phenolic fractions composition (µg g^−1^ FW) in selected four drought-tolerant leafy vegetables amaranth, different letters in the bar are differed significantly by Duncan multiple range test ((P < 0.01), (n = 3)).

Table 1 shows the data on main fragment ions in MS^2^, identified compounds, the molecular ion, λmax, and retention time. The liquid chromatography separated values of phenols and flavonoid compounds from four drought-tolerant leafy vegetable amaranth (VA16, VA14, VA11, and VA6) were compared with standard masses of phenols and flavonoid compounds through the respective peaks of the compounds. Twenty-five phenols and flavonoids compounds were determined in drought-tolerant leafy vegetable amaranth including nine benzoic acids, such as gallic acid, protocatechuic acid, gentisic acid, vanillic acid, *p*-hydroxybenzoic acid, salicylic acid, β-resorcylic acid, ellagic acid, and syringic acid; seven cinnamic acids, such as *m*-coumaric acid, caffeic acid, *trans*-cinnamic acid, ferulic acid, chlorogenic acid, sinapic acid, and *p*-coumaric acid; and nine flavonoids compounds, such as rutin, kaempferol, naringenin, isoquercetin, apigenin, myricetin, hyperoside, quercetin, and catechin. In our previous study, we also identified twenty-four flavonoids and phenolic acids in the leaves of red and green color amaranth^11^. Khanam et al.^54^ and Khanam and Oba^55^ noticed sixteen phenols and flavonoids compounds such as vanillic acid, syringic acid, gallic acid, salicylic acid, *p*-coumaric acid, *p*-hydroxybenzoic acid, rutin, *trans*-cinnamic acid, isoquercetin, caffeic acid, sinapic acid, *m*-coumaric acid, chlorogenic acid, ellagic acid, ferulic acid, and hyperoside in red and green amaranth. In the leaf, stalks, flowers, sprouts, and the seed of *A. cruentus*, *A. caudatus*, and *A. hypochondriacus*, Li et al.^56^ observed eleven phenolics including ferulic acid, chlorogenic acid, gallic acid, gentisic acid, β-resorcylic acid, protocatechuic acid, kaempferol, salicylic acid, rutin, quercetin, and ellagic acid. The cinnamic acids, gallic acid, vanillic acid, caffeic acids, *p*-hydroxybenzoic acid, *p*-coumaric acid, ferulic acid, syringic acids, and 3 flavonoids including isovitexin vitexin, and rutin were reported in the seeds and sprouts of *A. cruentus*^57^. Figures 1, 2, 3, and 4 showed the identified phenolic compounds of leaves of four selected drought-tolerant leafy vegetable amaranth. Across 3 main classes of phenolic compounds, the most identified pronounced compounds in four selected drought-tolerant leafy vegetable amaranth in the following order: flavonoids ˃ benzoic acids ˃ cinnamic acids.

**Table 1:** Retention time (Rt), wavelengths of maximum absorption in the visible region (λ_max_), mass spectral data and tentative identification of phenolic compounds in selected four drought-tolerant leafy vegetables amaranth.

Peak no	Rt (min)	λ_max_ (nm)	Molecular ion [M − H]^−^ (m/z)	MS^2^ (m/z)	Identity of tentative compounds
1	9.24	254	169.1224	169.1426	3,4,5 Trihydroxybenzoic acid
2	30.13	254	167.1121	167.1254	4-Hydroxy-3-methoxybenzoic acid
3	34.57	254	197.1231	197.1192	3,5-Dimethoxy-4-hydroxybenzoic acid
4	31.34	254	137.0224	137.1474	4-Hydroxybenzoic acid
5	48.23	254	137.2131	137.1832	2-Hydroxybenzoic acid
6	52.48	254	301.1432	301.0845	2,3,7,8-Tetrahydroxy-chromeno [5,4,3-cde] chromene-5,10-dione
7	2.33	280	154.1342	154.1256	3,4-Dihydroxybenzoic acid
8	4.28	280	154.1124	154.0256	2,4-Dihydroxybenzoic acid
9	3.82	280	154.1314	154.1258	2,5-Dihydroxybenzoic acid
10	32.12	280	179.0764	179.0875	3,4-Dihydroxy-trans-cinnamate
11	31.13	280	353.1426	353.1652	3-(3,4-Dihydroxy cinnamoyl) quinic acid
12	42.21	280	163.0584	163.1443	4-Hydroxy cinnamic acid
13	47.88	280	193.1691	193.1543	3-Methoxy-4-hydroxy cinnamic acid
14	49.59	280	163.2458	163.1273	3-Hydroxy cinnamic acid
15	49.03	280	223.1624	223.1642	4-Hydroxy-3,5-dimethoxy cinnamic acid
16	67.26	280	147.1214	147.1213	3-Phenyl acrylic acid
17	23.87	280	290.2387	290.1433	(2R-3S)-2-(3,4-dihydroxyphenyl)-3,4-dihydro-2-chromene-3,5,7-triol
18	26.71	280	271.0574	271.1248	Naringenin
19	54.33	360	463.2778	463.3234	Quercetin-3-*O*-glucoside
20	53.29	360	463.4435	463.5314	Quercetin-3-*O*-galactoside
21	53.14	360	609.2824	609.4123	Quercetin-3-*O*-rutinoside
22	7.54	370	301.1253	301.2138	2-(3,4-dihydroxy phenyl)-3,5,7-trihydroxychromene-4-one
23	4.58	370	626.3142	626.1362	Myricetin-3-*O*-rutinoside
24	15.55	370	270.2418	270.1317	4′,5,7-Trihydroxyflavone, 5,7-Dihydroxy-2-(4-hydroxyphenyl)-4-benzopyrone
25	17.77	370	593.4324	593.2847	kaempferol-3-*O*-rutinoside

### Benzoic acids

The most preponderant benzoic acids were identified as salicylic acids. Rest of the benzoic acids were identified in the order: gallic acid ˃ vanillic acid ˃ protocatechuic acid ˃ *p*-hydroxybenzoic acid ˃ gentisic acid ˃ β-resorcylic acid ˃ syringic acid ˃ ellagic acid (Fig. 1). It revealed from the present study that drought-tolerant leafy vegetable amaranth VA14 and VA16 exhibited much higher benzoic acid content in comparison with the results of the benzoic acid content of green amaranth of our previous study^11^ and the results of Khanam et al.^54^ in *A. tricolor*. The reason for higher benzoic acids obtained from our drought-tolerant vegetable amaranth genotypes in comparison with the results of our previous study and Khanam et al.^54^ may be due to differences in species, varieties, geographic locations, climatic and edaphic conditions, and cultural management. Salicylic acid, gallic acid, protocatechuic acid, gentisic acid, and β-resorcylic acid of selected drought-tolerant leafy vegetable amaranth varied from 9.37 to 23.61, 8.62 to 18.52, 3.55 to 15.53, 4.24 to 11.56, and 4.58 to 9.78 µg g^−1^ FW, respectively (Fig. 1). The highest salicylic acid (23.61 µg g^−1^ FW), gallic acid (18.52 µg g^−1^ FW), protocatechuic acid (15.53 µg g^−1^ FW), gentisic acid (11.56 µg g^−1^ FW), and β-resorcylic acid (9.78 µg g^−1^ FW) were obtained from the genotype VA14 followed by the genotype VA16. In contrast, the genotype VA6 showed the lowest salicylic acid (9.37 µg g^−1^ FW), gallic acid (8.62 µg g^−1^ FW), protocatechuic acid (3.55 µg g^−1^ FW), gentisic acid (4.24 µg g^−1^ FW), and β-resorcylic acid (4.58 µg g^−1^ FW). Vanillic acid, *p*-hydroxybenzoic acid, and ellagic acid ranged from 11.06 to 15.36, 2.58 to 12.33, and 1.24 to 6.23 µg g^−1^ FW, respectively (Fig. 1). The genotype VA16 exhibited the highest vanillic acid (15.36 µg g^−1^ FW), *p*-hydroxybenzoic acid (12.33 µg g^−1^ FW), and ellagic acid (6.23 µg g^−1^ FW) followed by VA14, while the genotype VA11 showed the lowest vanillic acid (11.06 µg g^−1^ FW) and the genotype VA6 exhibited the lowest *p*-hydroxybenzoic acid (2.58 µg g^−1^ FW), and ellagic acid (1.24 µg g^−1^ FW). The highest syringic acid was noticed in the genotype VA14 (7.63 µg g^−1^ FW) followed by VA16 and the lowest syringic acid was observed in the genotype VA14 (3.05 µg g^−1^ FW) (Fig. 1). Nine benzoic acids obtained in the current study were much higher than the results of nine cinnamic acids of green amaranth of our previous study^11^. The reason for higher benzoic acids obtained from our drought-tolerant vegetable amaranth genotypes in comparison with the results of our previous study may be due to differences in species.

### Cinnamic acids

*Trans*-cinnamic acid was identified as the most prominent component within cinnamic acids followed by chlorogenic acid. (Fig. 2). Selected drought-tolerant leafy vegetable amaranth had considerable cinnamic acids. The cinnamic acids obtained from the leafy vegetable amaranth genotype VA14 and VA16 were much higher in comparison with the results of cinnamic acids of green amaranth of our previous study^11^ and the results of *A. tricolor* reported by Khanam et al.^54^. The differences in species, varieties, geographic locations, climatic and edaphic conditions, and cultural management may be the reason for obtaining higher cinnamic acids from our drought-tolerant vegetable amaranth genotypes in comparison with the results of our previous study and Khanam et al.^54^. *Trans*-cinnamic acid, chlorogenic acid, sinapic acid, ferulic acid, and *p*-coumaric acid of selected drought-tolerant leafy vegetable amaranth varied from 8.56 to 19.88, 9.56 to 19.54, 8.94 to 16.35, 9.82 to 16.25, and 7.65 to 12.52 µg g^−1^ FW, respectively (Fig. 2). *Trans*-cinnamic acid, chlorogenic acid, sinapic acid, ferulic acid, and *p*-coumaric acid were the highest (19.88, 19.54, 16.35, 16.25, and 12.52 µg g^−1^ FW, respectively) in the genotype VA14 followed by the genotype VA16. However, the genotype VA6 exerted the lowest *trans*-cinnamic acid (8.56 µg g^−1^ FW), chlorogenic acid (9.56 µg g^−1^ FW), and *p*-coumaric acid (4.57 µg g^−1^ FW), while the genotype VA11 had the lowest sinapic acid (8.94 µg g^−1^ FW) and the genotype VA16 had the lowest ferulic acid (7.65 µg g^−1^ FW). Caffeic acid and *m*-coumaric acid ranged from 2.36 to 12.42 and 4.57 to 12.53 µg g^−1^ FW (Fig. 2). The highest caffeic acid and *m*-coumaric acid (12.42 µg g^−1^ FW and 12.53 µg g^−1^ FW) were reported in the genotype VA16 followed by the genotype VA14. In contrast, the genotype VA6 had the lowest caffeic acid and *m*-coumaric acid (2.36 µg g^−1^ FW and 4.57 µg g^−1^ FW). Seven cinnamic acids obtained in the current study were much higher than the results of seven cinnamic acids of green amaranth of our previous study^11^. The differences in species may be the reason for obtaining higher cinnamic acids from our drought-tolerant vegetable amaranth genotypes in comparison with the results of our previous study.

### Flavonoids

In the current investigation, selected drought-tolerant leafy vegetable amaranth had abundant flavonoids such as rutin, isoquercetin, quercetin, myricetin, naringenin, kaempferol, catechin, apigenin, and hyperoside which were much higher than the results of nine flavonoid compounds of green amaranth of our previous study^11^. The divergence in species may be the reason for obtaining higher flavonoids from our drought-tolerant vegetable amaranth genotypes in comparison with the results of our previous study. Rutin, isoquercetin, myricetin, and naringenin of selected drought-tolerant leafy vegetable amaranth varied from 17.29 to 46.56, 16.45 to 38.66, 9.38 to 25.56 and 4.74 to 22.75 µg g^−1^ FW, respectively (Fig. 3). The genotype VA14 exhibited the highest rutin, isoquercetin, myricetin, and naringenin (46.56, 38.66, 25.56, and 22.75 µg g^−1^ FW, respectively) followed y VA16, while the genotype VA6 showed the lowest rutin, isoquercetin, myricetin, and naringenin (17.29, 16.45, 9.38, and 4.74 µg g^−1^ FW, respectively). The highest quercetin was noticed in VA14 (32.32 µg g^−1^ FW) followed by VA11, while the genotype VA6 had the lowest quercetin content (10.62 µg g^−1^ FW). The genotype VA14 had the highest kaempferol (21.54 µg g^−1^ FW) followed by VA6, in contrast, the genotype VA11 exhibited the lowest kaempferol (12.18 µg g^−1^ FW) which was statistically similar to VA16 (12.36 µg g^−1^ FW). Catechin content was the highest in the genotype VA14 (21.53 µg g^−1^ FW) followed by VA6, while the genotype VA11 had the lowest catechin content (8.65 µg g^−1^ FW). The genotype VA14 and VA16 had high apigenin (8.85 and 7.75 µg g^−1^ FW), the genotype VA16 and VA14 had high hyperoside (6.38 and 5.66 µg g^−1^ FW). In contrast, the genotype VA11 showed the lowest apigenin and hyperoside (5.52 and 3.33 µg g^−1^ FW). (Fig. 3). Quercetin and hyperoside of our selected drought-tolerant leafy vegetable amaranth were higher than the content of quercetin and hyperoside reported by Khanam et al.^54^ in *A. tricolor* genotypes. The varietal differences, and differential geographic locations, climatic and edaphic conditions, and cultural managements may be played a major contribution in securing higher quercetin and hyperoside in our drought-tolerant vegetable amaranth genotypes in comparison with the results of Khanam et al.^54^.

### Phenolic fractions

Total flavonoids, total phenolic index, total phenolic acids, total benzoic acids, and total cinnamic acids of selected drought-tolerant leafy vegetable amaranth varied from 107.37 to 220.39, 51.76 to 114.75, 96.96 to 223.43, 55.61 to 105.64, and 204.33 to 443.82 µg g^−1^ FW, respectively (Fig. 4). The highest total phenolic acids (220.39 µg g^−1^ FW), total benzoic acids (114.75 µg g^−1^ FW), total phenolic index (443.82 µg g^−1^ FW), total cinnamic acids (105.64 µg g^−1^ FW), and total flavonoids (223.43 µg g^−1^ FW) were recorded in the genotype VA14 followed by the genotype VA16. In contrast, the lowest total cinnamic acids (55.61 µg g^−1^ FW), total benzoic acids (51.76 µg g^−1^ FW), total phenolic index (204.33 µg g^−1^ FW), total phenolic acids 107.37 µg g^−1^ FW), and total flavonoids (96.96 µg g^−1^ FW) were noticed in the genotype VA6 (Fig. 4). We noticed much greater total flavonoids total phenolic acids, and total phenolic index in selected leafy vegetables in comparison with the results of *A. tricolor* reported by Khanam et al.^54^. The varietal differences, and differential geographic locations, climatic and edaphic conditions, and cultural managements may be played a major contribution in securing higher phenolic fractions in our drought-tolerant vegetable amaranth genotypes in comparison with the results of Khanam et al.^54^. Cinnamic acid was synthesized in plant tissues from the most extensively distributed phenolic acids phenylalanine^58^. In the tissue of plants, although glycoside derivatives are the most common forms of flavonoids, occasionally these compounds occur as aglycone. Flavonoids represent approximately 60% of total dietary phenolic compounds^59^. The most predominant flavonoids in the plants are flavonols and quercetin glycosides are naturally occurring most prominent flavonols^59^. Significance differences in phenolic acids and flavonoids profiles among different *Cichorium spinosum* species were reported by Petropoulos et al.^60^.

In the current investigation, we observed abundant phenols and flavonoid compounds such as protocatechuic acid, salicylic acid, vanillic acid, gallic acid, β-resorcylic acid, *p*-hydroxybenzoic acid, naringenin, gentisic acid, myricetin, ellagic acid, chlorogenic acid, isoquercetin, syringic acid, *m*-coumaric acid, quercetin, caffeic acid, *trans*-cinnamic acid, rutin, sinapic acid, *p*-coumaric acid, catechin, ferulic acid, kaempferol, apigenin, and hyperoside in selected drought-tolerant leafy vegetable amaranth. We found corroborative results with the results of Khanam and Oba^55^ where they observed higher syringic acid, salicylic acid, *p*-hydroxybenzoic acid, vanillic acid, gallic acid, isoquercetin, ferulic acid, ellagic acid, rutin, *trans*-cinnamic acid, chlorogenic acid, *m*-coumaric acid, caffeic acid, and *p*-coumaric acid in red amaranth in comparison with green amaranth*. p*-hydroxybenzoic acid, rutin, *m*-coumaric acid, hyperoside, salicylic acid, chlorogenic acid, ferulic acid, ellagic acid, vanillic acid, gallic acid, syringic acid, caffeic acid, *trans*-cinnamic acid, and *p*-coumaric acid obtained from this study were higher than the results of Khanam et al.^54^ in *A. tricolor.* The selected drought-tolerant leafy vegetable amaranth VA14 and VA16 had high vitamins along with high flavonoids and phenols, such as protocatechuic acid, salicylic acid, gentisic acid, vanillic acid, gallic acid, *p*-hydroxybenzoic acid, β-resorcylic acid, ellagic acid, syringic acid, chlorogenic acid, *m*-coumaric acid, *trans*-cinnamic acid, caffeic acid, ferulic acid, *p*-coumaric acid, rutin, naringenin, sinapic acid, isoquercetin, myricetin, quercetin, kaempferol, catechin, apigenin, and hyperoside. The selected drought-tolerant leafy vegetable amaranth VA14 and VA16 could be used as phenolic profiles enrich high-yielding varieties. It revealed from the current investigation that these two genotypes containing high vitamins along with high flavonoids and phenols demand deep and elaborate pharmacological study to find the new insight of this crop.

### Antioxidant constituents and antioxidant capacity

**Figure 5:**
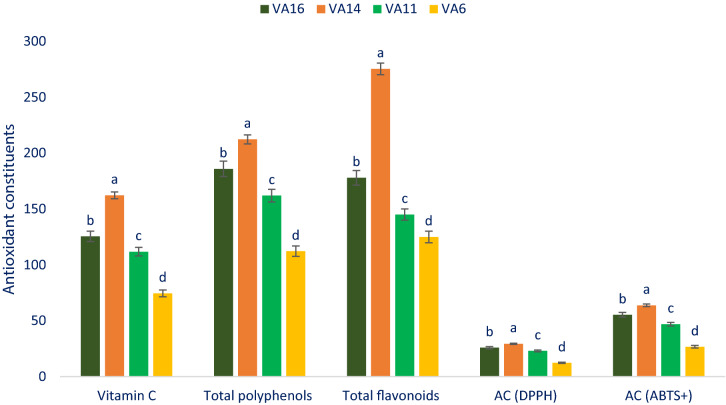
Antioxidant constituents and antioxidant capacity in selected four drought-tolerant leafy vegetables amaranth, Vitamin C (mg 100 g^−1^ FW), Total polyphenols (SAE µg g^−1^ DW), Total flavonoids (QE µg g^−1^ DW), *AC *(*DPPH*)  antioxidant capacity (DPPH) (TEAC µg g^−1^ DW), *AC* (*ABTS*^+^) antioxidant capacity (ABTS^+^) (TEAC µg g^−1^ DW), different letters in the bar are differed significantly by Duncan multiple range test ((P < 0.01), (n = 3)).

Total polyphenols, vitamin C, total flavonoids, and capacity of antioxidant (AC) varied remarkably among selected drought-tolerant leafy vegetable amaranth (Fig. 5). Vitamin C exhibited much remarkable variation in terms of genotypes, which varied from 74.45 mg 100 g^−1^ FW in the genotype VA6 to 162.34 mg 100 g^−1^ FW in the genotype VA14. Total polyphenols showed much remarkable variation in terms of genotypes with a range of 112.35 SAE µg g^−1^ DW (VA6) to 212.35 SAE µg g^−1^ DW (VA14). The accession VA14 showed the highest total phenols followed by VA16. Total flavonoids showed much remarkable variation in terms of genotypes, which varied from 125.00 QE µg g^−1^ DW in the genotype VA6 to 275.44 QE µg g^−1^ DW in the genotype VA14. Antioxidant capacity (DPPH) of selected drought-tolerant leafy vegetable amaranth varied from 12.27 TEAC µg g^−1^ DW (VA6) to 29.38 TEAC µg g^−1^ DW (VA14). The highest antioxidant capacity (DPPH) was recorded in the genotype VA14 followed by VA16 and VA11. On the other hand, the lowest antioxidant capacity (DPPH) was noticed in VA6. Antioxidant capacity (ABTS^+^) of selected drought-tolerant leafy vegetable amaranth varied from 26.69 TEAC µg g^−1^ DW to 63.79 TEAC µg g^−1^ DW. The selected vegetable amaranth VA14 had the highest antioxidant capacity (ABTS^+^) followed by VA16. On the other hand, antioxidant capacity (ABTS^+^) was the lowest in VA6. The current findings were corroborative with the results of Khanam and Oba^55^ where they noticed higher total flavonoids, total antioxidant capacity, and total polyphenols in red amaranth in comparison with green amaranth*.* The selected drought-tolerant leafy vegetable amaranth VA14 and VA16 contained higher vitamin C, total flavonoids, total polyphenols, and antioxidant capacity in comparison with VA11 and VA6. Hence, these antioxidant constituents of leafy vegetable amaranth could be crucial attributes for consumers due to the high detoxifying capacity of ROS in the human body and preventing many degenerative human diseases and anti-aging activity^18,20^. It suggested from the present results of vitamin C, total flavonoids, total polyphenols, and antioxidant capacity in selected drought-tolerant leafy vegetable amaranth that leafy vegetables have important free radical-scavenging activity^21^.

In the current investigation, we observed remarkable vitamin C, total flavonoids, total polyphenols, and antioxidant capacity in selected drought-tolerant leafy vegetable amaranth. The current results were corroborative with the results of total polyphenols, total flavonoids, and antioxidant capacity of Khanam and Oba^55^. They noticed higher antioxidant capacity, total flavonoids, and total polyphenols content in *A. tricolor* genotypes in comparison with green amaranth genotype*.* Antioxidant capacity (ABTS^+^), total flavonoids and antioxidant capacity (DPPH) obtained in the current study were corroborated with the results of *A. tricolor* reported by Khanam et al.^54^, while total phenols noticed in this investigation was much pronounced than total phenols in *A. tricolor* reported by Khanam et al.^54^*.* Vitamin C obtained from our study was much greater than vitamin C reported by Jiminez-Aguilar and Grusak^61^ in *Amaranthus* species. The varietal differences, and differential geographic locations, climatic and edaphic conditions, and cultural managements may be played a key role in accumulating higher phenols and vitamin C in our drought-tolerant vegetable amaranth genotypes in comparison with the results of Khanam et al.^54^ and Jiminez-Aguilar and Grusak^61^. The higher total antioxidant activity (FRAP and ORAC methods), total flavonoids, and total phenols were reported in the leaves of *A. hypochondriacus* than *A. caudatus* leaves^56^. They reported the highest total antioxidant activity (FRAP), total flavonoids, and total phenols in the leaves than stalks, seed, flowers, and sprouts. All the extraction and estimation methods and standard references differed to our methodology, hence, it is tedious to compare our present results with their results. The genotypes VA14 and VA16 had high phenolic profiles, antioxidant constituents such as vitamin C, total polyphenols, total flavonoids, and antioxidant capacity. The selected drought-tolerant leafy vegetable amaranth VA14 and VA16 could be used as antioxidant profiles enrich high-yielding varieties. It revealed from the study that these two genotypes could offer greatly contributed to feeding the antioxidant-deficient community.

### Correlation coefficient study

The correlation of antioxidant constituents and antioxidant capacity of selected drought-tolerant leafy vegetable amaranth are shown in Table 2. Vitamin C had significant positive interrelationships with total flavonoids, total polyphenols, and antioxidant capacity (DPPH and ABTS^+^) that signify that vitamin C had high antioxidant activity. The results of the present study corroborated with the results of our earlier study of drought and salt-stressed *A. tricolor*^22–24,28^. The significant correlation among total polyphenols, total flavonoids, antioxidant capacity (DPPH and ABTS^+^) were observed indicating potential antioxidant activity of total polyphenols and total flavonoids of selected drought-tolerant leafy vegetable amaranth. The findings for total antioxidant capacity (FRAP), total flavonoids, and total polyphenols in salt-stressed purslane^62^ were corroborative to our present findings. Similarly, AC (ABTS^+^) significantly associated with AC (DPPH) that validated the estimation of antioxidant activity of two different methods in selected leafy vegetables.

**Table 2:** The correlation coefficient for antioxidant constituents, and antioxidant capacity in selected four drought-tolerant leafy vegetables amaranth.

	Total polyphenols (GAE µg g^−1^ FW)	Total flavonoids (RE µg g^−1^ DW)	AC (DPPH) (TEAC µg g^−1^ DW)	AC (ABTS^+^) (TEAC µg g^−1^ DW)
Vitamin C	0.86**	0.89**	0.86**	0.92**
Total polyphenols		0.87**	0.92**	0.95**
Total flavonoids			0.84**	0.87**
AC (DPPH)				0.98**

*AC* (*DPPH*)  antioxidant capacity (DPPH), *AC* (*ABTS*^+^) antioxidant capacity (ABTS^+^).

*Significant at 5% level.

**Significant at 1% level, (n = 3).

In conclusion, we identified twenty-five phenols and flavonoid compounds such as protocatechuic acid, *p*-hydroxybenzoic acid, vanillic acid, salicylic acid, gentisic acid, β-resorcylic acid, gallic acid, ellagic acid, chlorogenic acid, syringic acid, *m*-coumaric acid, caffeic acid, *trans*-cinnamic acid, ferulic acid, *p*-coumaric acid, sinapic acid, naringenin, isoquercetin, rutin, kaempferol, hyperoside, catechin, apigenin, myricetin, and quercetin in selected drought-tolerant leafy vegetable amaranth. The selected leafy vegetable amaranth VA14 and VA16 exhibited remarkable phenols, vitamins, flavonoids, antioxidant constituents, and antioxidant potentiality. It revealed from the correlation study that all antioxidant compositions of selected drought-tolerant leafy vegetable amaranth exhibited high antioxidant potentiality. It revealed from the study that two selected drought-tolerant leafy vegetable amaranth showed excellent sources of antioxidants components including high ROS quenching capacity that offered huge prospects for attaining antioxidant sufficiency in the world. It revealed from this study that data reported from selected drought-tolerant leafy vegetable amaranth greatly contributed to the scientists to evaluate pharmacologically active constituents.

## Methods

### Experimental materials

It is the first report on phenolic profiles, antioxidant compositions, and antioxidant capacity in drought-tolerant leafy vegetable amaranth. We previously evaluated 43 genotypes for antioxidant and yield potentiality to select the best four high yielding and antioxidant enrich genotypes for this experiment.

### Design and layout

We executed the experiment in three replicates following a completely randomized block design (RCBD) at Bangabandhu Sheikh Mujibur Rahman Agricultural University. Each genotype was grown in 1 m^2^ experimental plot following 20 cm and 5 cm distance between rows and plants, respectively.

### Intercultural practices

Recommended compost doses, fertilizer, and appropriate cultural practices were maintained^63^. For maintaining the exact spacing of plants in a row, proper thinning was executed. Weeds of experimental plots were regularly removed through proper weeding and hoeing. We provide regular irrigation in the experimental plots for maintaining the proper growth of vegetable amaranth. We collected the leaf samples at 30 days old plant.

### Solvents and reagents

Methanol, acetic acid (HPLC grade), acetonitrile (HPLC grade), acetone, standard phenolic compounds, 2, 2-dipyridyl, dithiothreitol (DTT), DPPH (2, 2-diphenyl1-picrylhydrazyl), standard Trolox, ABTS^+^, gallic acid, aluminum chloride hexahydrate, folin-ciocalteu reagent, potassium acetate, rutin, sodium carbonate, and potassium persulfate. All solvents and reagents were bought from Merck (Germany) and Kanto Chemical Co. Inc. (Tokyo, Japan).

### Samples extraction for HPLC and LC–MS analysis

The leaf samples were extracted by adding 10 ml methanol (80%) containing acetic acid (1%) in 1 g leaves. The mixture was thoroughly homogenized. Then the mixture was kept to a test tube (50 ml) and capped tightly. The test tube was shaken in a shaker (Scientific Industries Inc., USA) for 15 h at 400 rpm. Exactly 0.45 µm filter (MILLEX-HV syringe filter, Millipore Corporation, Bedford, MA, USA) was used to filter the homogenized mixture. We centrifuged the mixture at 10,000 × *g* for 15 min. The phenolic compounds were analyzed from the final filtrate. We performed all extractions in triplicate independent samples.

### Flavonoids, and phenolic acids analysis through HPLC

The method previously described by Sarker and Oba^11,28^ was followed to phenolic profile, respectively in leaf sample using HPLC. We equipped the Shimadzu SCL10Avp (Kyoto, Japan) HPLC with a binary pump (LC-10Avp), DGU-14A degasser, and a Shimadzu SPD-10Avp UV–vis detector. A CTO-10AC (STR ODS-II, 150 × 4.6 mm I.D., (Shinwa Chemical Industries, Ltd., Kyoto, Japan) column was used for the separation of flavonoids and phenolic acids^11^. The binary mobile phase was pumped with solvent A (6% (v/v) acetic acid) in water and solvent B (acetonitrile) at the flow rate of 1 ml/min for 70 min. HPLC system was run using a gradient program with 0–15% acetonitrile for 45 min, 15–30% for 15 min, 30–50% for 5 min, and 50–100% for 5 min. 35 °C temperature in the column was maintained with a 10 μl volume of injection^11^. We set the detector at 360, 370, 280, and 254 nm, respectively for continuous monitoring of flavonoids, cinnamic acids, and benzoic acids. For identification of the compound, we compared retention time and UV–vis spectra with their respective standards. We confirmed the flavonoids, and phenolic acids through the mass spectrometry assay method. HPLC detected total compounds were represented as a total phenolic index (TPI). The previously described method of Sarker and oba^11,28^ was used to TPI from the HPLC data. All samples were prepared and analyzed in duplicate. We estimated phenolic compounds as µg g^−1^ FW. A mass spectrometer (AccuTOF JMS-T100LP, JEOL Ltd., Tokyo, Japan) fitted with an Agilent 1100 Series HPLC system and a UV–vis detector coupled on-line with an ElectroSpray Ionization (ESI) source to analyze the mass spectrometry with negative ion mode with the column elutes in the range of m/z 0–1,000 and needle voltage at – 2,000 V. Extract constituents were identified by LC–MS-ESI analysis.

### Quantification of phenolic compounds

We used the respective standards of calibration curves to quantify each phenolic compound. We dissolved 25 phenolic compounds in 80% methanol as stock solutions to the final concentration of 100 mg/ml. Respective standard curves (10, 20, 40, 60, 80, and 100 mg/ml) were used to quantify the individual phenolic compounds with external standards. UV spectral characteristics, retention times and co-chromatography of samples spiked with commercially available standards were utilized for identification and match the phenolics.

### Estimation of vitamin C

A Hitachi spectrophotometer (U-1800, Tokyo, Japan) was utilized to estimate ascorbic acid (AsA) and dehydroascorbic acid (DHA) from the fresh amaranth leaves. Dithiothreitol (DTT) was used for the sample pre-incubation and reduction of dehydroascorbic acid into ascorbic acid. Ascorbic acid reduced ferric ion to ferrous ion. Reduced ferrous ion forms complexes with 2, 2-dipyridyl^8^. We read the absorbance of Fe_2_^+^ complexes with 2, 2-dipyridyl at 525 nm for estimation of vitamin C through the spectrophotometric (Hitachi, U-1800, Tokyo, Japan). We calculated vitamin C in mg 100 g^−1^ FW.

### Estimation of total polyphenols

Extraction of total polyphenols was carried out according to Sarker and Oba^64^ using 25 mg of sample in 2.5 mL of 1.2 M HCl containing methanol (90%) at 90 °C for 2 h in a water bath. With readjusting the volume (2.5 mL), the leaf extract was centrifuged at 7,500 rpm for 20 min. The leaf extracts (100 µL) were added to the Folin–Ciocalteau reagent (2 N, 50 µL). After 5 min, 2 N Na_2_CO_3_ (400 µL) and water (1 mL) was added. The leaf extracts were incubated for 90 min at 37 °C. Finally, it was removed to a microplate (flat bottom). In a microplate reader, the absorbance was detected at 740 nm. We estimated the results in equivalent to gallic acid (GAE) standard µg g^−1^ of FW.

### Estimation of total flavonoids

Total flavonoids were extracted and quantified according to the method described by Sarker and Oba^65^. Samples (100 mg) were mixed with 5 mL methanol (50%) in water and placed for 1 h with ultrasound. The leaf extracts were centrifuged for 10 min at 13,000 *g* (4 °C). The supernatants were then recovered. Flavonoid extracts (400 µL) were homogenized with water (500 µL), 5% NaNO_2_ (60 µL), 10% AlCl3 (140 µL). After 10 min, 1 mM NaOH (400 µL) was added. The leaf extracts were incubated for 10 min at a normal temperature. Finally, it was removed to a flat bottom microplate. The absorbance was read at 500 nm in a microplate reader. Results are expressed in µg of rutin equivalents (RE) per gram of sample DW.

### Radical quenching capacity assay

Thirty days old amaranth leaves were harvested. Antioxidant capacity assay, the leaves were dried in the air in a shade. 40 ml aqueous methanol (90%) was utilized to extract grounded dried leaves (1 g) from each cultivar in a capped bottle (100 ml). A Thomastant T-N22S (Thomas Kagaku Co. Ltd., Japan) shaking water bath was utilized to extract leaf samples for 1 h. Exactly 0.45 µm filter (MILLEX-HV syringe filter, Millipore Corporation, Bedford, MA, USA) was used to filter the homogenized mixture. After centrifugation for 15 min at 10,000 × *g*, the antioxidant capacity was estimated from the filtered extract.

Diphenyl-picrylhydrazyl (DPPH) radical degradation method^66–68^ was used to estimate the antioxidant activity. We added 1 ml DPPH solution (250 µM) to 10 µl extract (in triplicate) in a test tube. After adding 4 ml distilled water the extract was placed in the dark for 30 min. A Hitachi U1800 spectrophotometer (Hitachi, Tokyo, Japan) was used to measure the absorbance at 517 nm. Method of Sarker and Oba^28^ was followed for ABTS^+^ assay. To prepare two stock solutions separately ABTS^+^ solution of 7.4 mM and potassium persulfate of 2.6 mM were used. We mixed both solutions in equal proportion to prepare the working solution at room temperature. The working solution was allowed to react in the dark for 12 h. One hundred fifty μl extract was added to 2.85 ml of ABTS^+^ solution and allowed to react in the dark for 2 h. For the preparation of the solution, one ml of ABTS^+^ solution was mixed with sixty ml of methanol. A Hitachi spectrophotometer (U1800, Tokyo, Japan) was utilized to take the absorbance against methanol at 734 nm. The inhibition (%) of DPPH and ABTS^+^ corresponding with control was used to determine antioxidant capacity using the equation as follows:

$$ {\text{Antioxidant activity }}\left( \% \right) \, = \, \left( {{\text{Abs}}.{\text{ blank}} - {\text{ Abs}}.{\text{ sample}}/{\text{Abs}}.{\text{ blank}}} \right) \, \times { 1}00 $$

where, Abs. blank is the absorbance of the control reaction [10 µl methanol for TAC (DPPH), 150 μl methanol for TAC (ABTS^+^) instead of leaf extract] and Abs. sample is the absorbance of the test compound. Trolox was used as the reference standard, and the results were expressed as μg Trolox equivalent g^−1^ DW.

### Statistical analysis

Statistix 8 software was used to analyze the data for analysis of variance (ANOVA)^69,70^. Duncan’s Multiple Range Test (DMRT) at a 1% level of probability was used to compare the means. The results were reported as the mean ± SD of three separate replicates.

### Ethical statement

The lab and field experiments in this study were carried out as per guidelines and recommendations of “Biosafety Guidelines of Bangladesh” published by the Ministry of Environment and Forest, Government of the People’s Republic of Bangladesh (2005).

## Data Availability

Data used in this manuscript will be available to the public.
